# Current and future advances in practice: aromatase inhibitor–induced arthralgia

**DOI:** 10.1093/rap/rkae024

**Published:** 2024-04-10

**Authors:** Sara Kim, Nan Chen, Pankti Reid

**Affiliations:** Department of Medicine, University of Chicago, Chicago, IL, USA; Division of Hematology and Oncology, Department of Medicine, University of Chicago, Chicago, IL, USA; Division of Rheumatology, Department of Medicine, University of Chicago, Chicago, IL, USA

**Keywords:** aromatase inhibitor, arthralgia, breast cancer, aromatase inhibitor–associated joint pain, arthritis

## Abstract

Aromatase inhibitors (AIs) have shown great success as adjuvant therapy for post-menopausal women with hormone receptor–positive breast cancers. AI-induced arthralgia (AIA) is a frequent AI toxicity contributing to non-adherence and discontinuation. This review aims to understand current knowledge of AIA. The mean incidence of AIA was 39.1% and the mean discontinuation of AI therapy due to AIA was 9.3%. Most of the AIAs were non-inflammatory. A shorter time since the last menstrual period and pre-existing joint pain were risk factors. Vitamin D3 supplementation may be a preventative measure and treatment with duloxetine, acupuncture and/or exercise is supported by large randomized controlled trials. There was consistent improvement in AIAs with switching to an alternate AI, and this could additionally allow continuation of cancer treatment with AI. Further research is needed to identify predictive biomarkers, better characterize AIA subcategories and study more reliable therapeutic options.

Key messages

- AIA contributes to non-adherence and discontinuation of AI.
- Most AIAs are not inflammatory in character.
- Duloxetine, acupuncture and physical activity such as yoga, exercise and tai chi can alleviate AIA symptoms.
- Changing to a different type of AI can lead to resolution or attenuation of AIA symptoms.
- Larger randomized controlled trials are needed on therapeutic and preventative methods.

## Introduction

Breast cancer is the most commonly diagnosed malignancy in women, and 70% are hormone receptor positive (HR^+^). Current treatment guidelines recommend aromatase inhibitors (Ais) as adjuvant therapy for post-menopausal women with HR^+^ breast cancers [1]. AIs are the preferred treatment because AIs have prolonged disease-free survival compared with the selective oestrogen receptor modulator tamoxifen [2]. Despite their proven efficacy, AIs have been associated with discontinuation rates as high as 32.4% [3–5], with adverse effects being the most common reason for drug discontinuation [6]. With the latest guideline recommending extended AI therapy up to 10 years in high-risk disease, reducing AI toxicity and achieving the greatest drug tolerance should be a high clinical priority [1, 7]. Of all the AI toxicities, joint pain is noted to be the most common reason for ceasing AI therapy [6], with an estimated prevalence of 46% [8]. Joint pain that develops or worsens after AI therapy initiation is grouped together under the terminology AI-induced arthralgia (AIA) [9]. AIA contributes to non-adherence and premature AI discontinuation, both of which are associated with higher breast cancer recurrence rates and worsened mortality [10, 11].

Given the lack of clinical guidelines on diagnosis, prevention and management of AIA symptoms, our review aims to better elucidate the current understanding of AIA and provide insights to upcoming clinical and research developments within this field.

## Methods

A literature search was performed from the MEDLINE/PubMed database and studies published up to March 2023 were included. Search terms included ‘aromatase inhibitor induced arthralgia’, ‘arthritis’, ‘anastrozole’, ‘letrozole’ and ‘exemestane’. Initial screening was done based on the titles and abstracts, and any articles with a primary focus of ‘joint pain’, ‘joint stiffness’, ‘AI-induced musculoskeletal symptoms’, ‘AI-associated arthralgia’, ‘arthritis’ or ‘AI-induced arthralgia’ were included. In-depth full-text screening was performed and the types of studies included were letters to editors with original data, case reports, case series, prospective cohort studies, retrospective cohort studies and randomized controlled trials (RCTs). Any studies that did not address AI-induced arthralgia in breast cancer patients, feasibility studies, pilot studies in which a more recent version was available, reviews, correspondence articles, letters to editors without original data and duplicate studies were excluded. Screening of abstracts and titles was performed using Sciwheel (Technology from Sage, London, UK). Descriptive statistics were used for data analysis.

## Results

**Figure 1.**
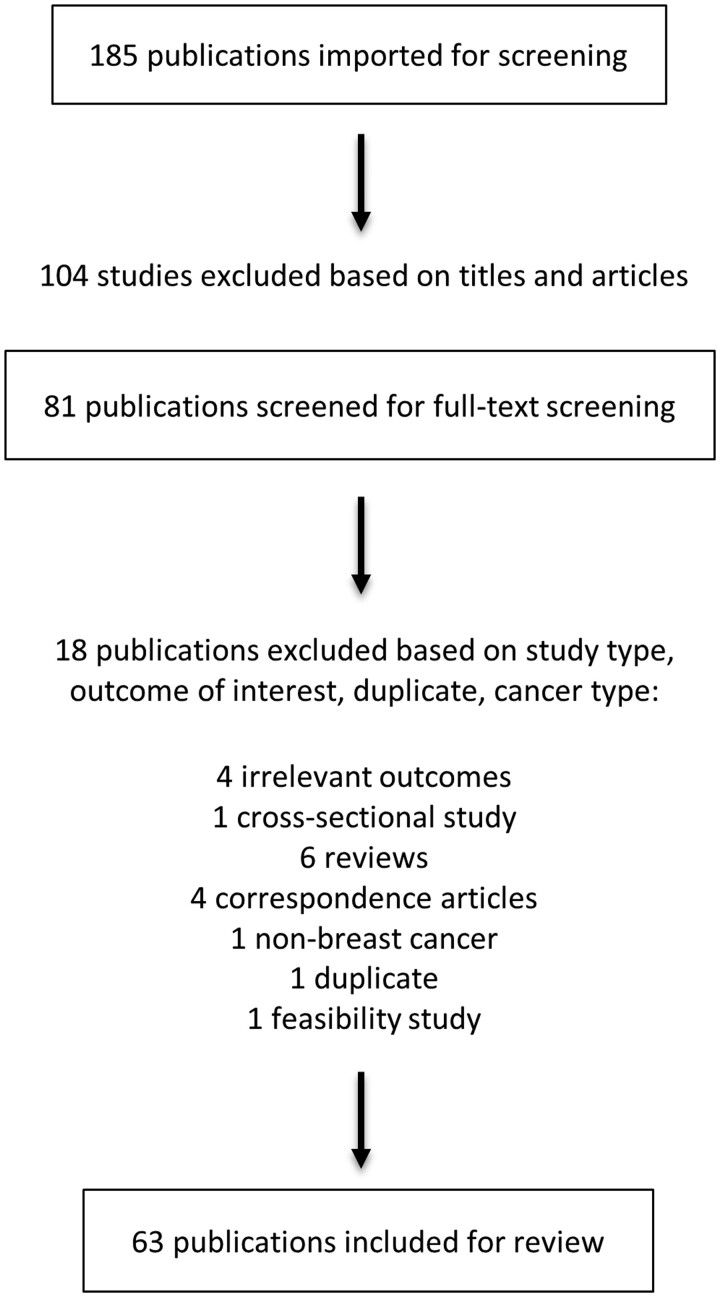
Of the 185 publications screened, 122 were excluded according to the exclusion criteria and ultimately 63 publications were selected for the review

Of the 185 publications screened, 122 were excluded according to the exclusion criteria. From the 63 publications selected for the review, 15 were prospective RCTs, 33 were prospective observational studies, 12 were retrospective observational studies and 3 were case reports (Fig. 1).

### Characteristics of arthralgia

Among 23 publications that reported the incidence of AIA (Table 1), the mean incidence of AIA was 39.1%. Among studies reporting the number of joints affected, four of five studies reported the mean number of joints affected to be oligoarticular (fewer than five joints) whereas one study reported polyarticular (more than five joints). The most commonly affected joints were the hands/wrists, knees, back, hips, ankles/feet and others (shoulders, elbows, neck), in descending order. The mean time to onset of AIA was 4.5 months after AI initiation. The most frequently used methods of assessment of arthralgia were the Brief Pain Inventory (BPI), Western Ontario and McMaster Osteoarthritis Index (WOMAC), visual analogue scale (VAS), Modified Score for the Assessment of Chronic Rheumatoid Affections of the Hands (M-SACRAH) and self-administered questionnaire (specifically designed for the study), in descending order (Table 2).

**Table 1.** Characteristics of AIA

Reference (23 total studies)	Type of study	Subjects on AI, *n* (20 652 total patients)	Incidence of AIA, % (*n*/*N*)	Affected joints, *n* and type(s)	Time to onset of AIA	Discontinuation of AI due to AIA, % (*n*/*N*)
Sagara *et al*., 2010 [12]	PO	656	3.6 (24/656)	Oligoarticular^a^; NR	After 6–18 months	NR
Shi *et al*., 2013 [13]	PO	47	34 (16/47)	Oligoarticular; knees, hands	Mean 2.2 at 3 months, median 1.6 months	NR
Presant *et al*., 2007 [14]	PO	56	61 (34/56)	NR; peripheral (79%), elbow (59%), central (50%), knee, wrist, feet (44%), spine (32%), shoulder, hip (26%)	Mean 1.7 months	19.6 (11/56)
Park *et al*., 2013 [15]	RO	299	23 (69/299)	NR; NR	Within 6 months	9.7 (29/299)
Chien *et al*., 2020 [16]	RO	4038	13 (525/4038)	NR; NR	NR	NR
Boonstra *et al*., 2013 [17]	PO	57	74 (42/57)	Polyarticular^b^; fingers, hips, knees	NR	NR
Horimoto *et al*., 2009 [18]	RO	329	27 (90/329)	NR; NR	Median 6 months	0.3 (1/329)
Honma *et al*., 2019 [19]	PO	2058 patients, 8385 survey responses	44.3 (3716/8385)	NR; NR	NR	NR
Shanmugam *et al*., 2011 [20]	RO	*N* = 48; AI group *n* = 25, control group *n* = 23	68 (17/25)	NR; NR	Mean 10.4 months	NR
Laroche *et al*., 2014 [21]	PO	135	36 (48/135)	NR; NR	NR	NR
Hadji *et al*., 2012 [22]	RO	1502	30.8 (235/763) at 30 months, 23 (176/763) after 30 months	NR; NR	NR	NR
Zhu *et al*., 2022 [23]	PO	*N* = 155; AI group *n* = 49, control group *n* = 106	NR	NR; knees (28%), lower back (26%), ankles/feet (17%)	NR	NR
Kuria *et al*., 2012 [24]	RO	1400	14 (200/1400)	NR; hands/wrist (62%), knee (60%), back (40%), hip (37%)	Mean 3 months	NR
Mao *et al*., 2009 [25]	PO	300	47 (139/300)	Oligoarticular; wrist/hand (60.4%), knee (59.7%), back (54.0%), ankle/foot (51.8%) and hip (42.5%)	Majority (75%) within 3 months	NR
Nabieva *et al*., 2019 [26]	PO	1416	NR	NR; NR	Within 6 months	NR
Robidoux *et al*., 2011 [27]	PO	30	17 (5/30)	NR; NR	After 1.6 months	NR
Moxley, 2010 [28]	PO	*N* = 77; AI group *n* = 36, control group *n* = 41	50 (18/36)	Oligoarticular; hands, knees, neck, shoulders	NR	NR
Castel *et al*., 2013 [29]	PO	*N* = 268; AI group *n* = 91, control group *n* = 177	NR	NR; NR	NR	NR
Fontaine *et al*., 2008 [30]	RO	185	45 (83/185)	NR; NR	NR	12.4 (23/185)
Garcia-Giralt *et al*., 2013 [31]	PO	343	48.2 (161/334) at 3 months, 54.8 (176/321) at 12 months	NR; NR	NR	4.4 (15/343)
Kanematsu *et al*., 2011 [32]	PO	328	34.8 (114/328)	NR; NR	Peaks at 4 months (33.7%) and 8 months (11.4%)	NR
Egawa *et al*., 2016 [33]	PO	362	71.8 (260/362)	NR; NR	Mean 5.4 months	NR
Moscetti *et al*., 2015 [34]	RO	236	26.6 (62/236)	NR; NR	Median 9.1 months	5.5 (13/236)
Mean (SD)		623 (933)	39.1 (19.5)	Hands/wrists 61.2% (0.8), knees 49.2% (15.0), back 38% (10.5), hip 35.2% (6.9), ankles/feet 34.4% (17.4)	4.5 months (3.2)	9.3 (6.6)

NR: not reported; PO: prospective observational study; RO: retrospective observational study.

a oligoarticular: <5 joints; ^b^polyarticular: >5 joints.

**Table 2.** Methods of assessment

Assessment of arthralgia	Studies, *n*
Brief Pain Inventory (BPI)	22
Western Ontario and McMaster Osteoarthritis Index (WOMAC)	17
Visual analogue scale (VAS)	7
Modified Score for the Assessment of Chronic Rheumatoid Affections of the Hands (M-SACRAH)	6
Self-administered questionnaire	5
Australian/Canadian Hand Osteoarthritis Index (AUSCAN)	3
Disabilities of the Arm, Shoulder and Hand (DASH)	3
Numeric rating scales (0–10)	2
MD Anderson Symptom Inventory (MDASI)	2
Pain Disability Index (PDI)	1
Rheumatoid Arthritis Disease Activity Index (RADAI)	1
Dutch Arthritis Impact Measurement Scales (AIMS)	1
Breast Cancer Prevention Trial Symptom Scale–Musculoskeletal Subscale (BCPT-MS)	1
Modified BPI for Aromatase Inhibitor Arthralgia (BPI-AIA)	1
Outcome Measures for Arthritis Clinical Trials–Osteoarthritis Research Society International (OMERACT-OARSI)	1
Arthritis Impact Measurement Scale (AIMS2)	1
Patient-Reported Arthralgia Inventory (PRAI)	1
Joint-pain questionnaire (JPA)	1
Pain Severity Index (PSI)	1
28-joint Disease Activity Score (DAS-28)	1

### Inflammatory arthritis

According to Shanmugam *et al.* [20], no significant differences were seen in 28-joint DAS (DAS28) scores or inflammatory markers between those who developed AIA and controls, indicating a predominantly non-inflammatory aetiology for patient’s joint pain. In the same study, two patients had a diagnosis of RA and two with Sjogren’s disease prior to AI initiation; however, the same number of patients with autoimmune diseases was found in the group who developed AIA and the group who did not, indicating that pre-existing autoimmune diseases were not consistently associated with development of AIA [20].

### Discontinuation due to arthralgia

The mean percentage of subjects who discontinued AI therapy due to AIA was 9.3%. One of the studies reported that 10 of 17 (58.8%) patients with AIA experienced resolution of the symptoms on average 3 months after cessation and 2 of 17 (11.8%) patients experienced a >50% reduction in pain scores [35].

### Risk factors for AIA

#### Impact of menopause and menstruation

As reported in Table 3, a shorter time since the last menstrual period (<5 years) [25, 32], a shorter time since menopause [33] and worse menopausal symptoms at baseline [29] were associated with a higher incidence of AIA (all three factors had *P*-values <0.05).

**Table 3.** Risk factors of AIA

	Risk factors	Subjects on AI, *n*	Type of study	Reference
Impact of menstruation and menopause	Greater incidence in patients with <5 years since LMP *vs* >10 years since LMP (*P* = 0.02)	300	PO	Mao *et al*., 2009 [25]
	Greater incidence in patients with short time since menopause (12.5 years) *vs* greater time since menopause (16.4 years) (*P* = 0.02)	391	PO	Egawa *et al*., 2016 [33]
	Greater severity in patients with greater severity of menopausal symptoms at baseline (*P* = 0.04)	91	PO	Castel *et al*., 2013 [29]
	Greater incidence in patients with <5 years since LMP *vs* >10 years since LMP (*P* = 0.002). Earlier manifestation of AIA (≤6 months) in patients with <5 years since LMP *vs* >10 years since LMP (*P* = 0.036)	328	PO	Kanematsu *et al*., 2011 [32]
Pre-existing comorbidities	Greater severity in patients with pre-existing joint-related comorbidities (OA, RA, PsA, lupus, gout, AS, FM, osteoporosis, osteopenia or SS) (*P* = 0.01)	91	PO	Castel *et al*., 2013 [29]
	Greater incidence in patients with higher BMI (*P* < 0.05)	57	PO	Boonstra *et al*., 2013 [17]
	Greater increase in pain values during the first 6 months of AI therapy in patients without pre-existing joint pain *vs* with pre-existing joint pain (*P* < 0.00001)	1416	PO	Nabieva *et al*., 2019 [26]
	Greater incidence in patients with older age [>75 years *vs* <45 years; aHR = 3.18 (95% CI 2.65, 3.81)], hypertension [aHR 1.11 (95% CI 1.02, 1.2)], dyslipidaemia [aHR 1.18 (95% CI 1.06, 1.30)] or affective disorders [aHR 1.32 (95% CI 1.23, 1.43)]	4038	RO	Chien *et al*., 2020 [16]
	Greater incidence in patients with pre-existing pain (*P* = 0.036) and in patients with BPI worst pain score ≥1 (*P* = 0.018)	47	PO	Shi *et al*., 2013 [13]
	Greater incidence of osteopenia and osteoporosis in patients with AIA (*P* < 0.001)	316	RO	Muslimani *et al*., 2009 [36]
Previous treatments	Greater incidence of AI discontinuation due to AIA in patients with prior use of tamoxifen (*P* < 0.01)	299	RO	Park *et al*., 2013 [15]
	Greater incidence in patients with prior receipt of radiation therapy [aHR 1.16 (95% CI 1.05, 1.27)], opioid use [aHR 1.37 (95% CI 1.19, 1.59)] and NSAIDs/acetaminophen use [aHR 1.32 (95% CI 1.19, 1.47)]	4038	RO	Chien *et al*., 2020 [16]
	Greater incidence in patients with prior receipt of adjuvant chemotherapy (*P* = 0.03)	391	PO	Egawa *et al*., 2016 [33]
Type of AI	Greater incidence with letrozole use *vs* tamoxifen use [aHR 1.27 (95% CI 1.12, 1.4)]	4038	RO	Chien *et al*., 2020 [16]
	Greater incidence in patients who received steroidal AIs *vs* non-steroidal AIs (*P* < 0.001)	316	RO	Muslimani *et al*., 2009 [36]
Genetic factors	Greater incidence in patients with SNPs in the *CYP17A1* and *VDR* genes (*P* = 0.003 and *P* = 0.012, respectively). Association of AI discontinuation in patients with SNPs in the *CYP27B1* gene (*P* = 0.02)	343	PO	Garcia-Giralt *et al*., 2013 [31]
	Greater incidence (*P* = 0.046) and severity of pain (*P* = 0.018) in patients with the G allele of rs2073618 in OPG *vs* wild-type	159	PO	Lintermans *et al*., 2016 [37]

aHR: adjust hazard ratio; LMP: last menstrual period; OPG: osteoprotegerin; SNP: single-nucleotide polymorphism; VDR: vitamin D receptor.

#### Pre-existing comorbidities

Pre-existing affective disorders, older age (e.g. >75 years), hypertension, dyslipidaemia and baseline joint pain were statistically significant predictors of development of AIA [13, 16, 21, 26]. In addition, patients with AIA had higher incidences of higher BMI [17] and osteopenia/osteoporosis [36]. Furthermore, one study reported a significant association with joint-related comorbidities such as osteoarthritis, RA, PsA, lupus, gout, AS, FM, osteoporosis and SS with increased AIA severity [29].

#### Previous treatments

Prior opioids, NSAIDs or acetaminophen use were significant risk factors for AIA, as was prior receipt of adjuvant chemotherapy or radiation therapy [16, 33]. Prior tamoxifen use was specifically associated with discontinuation of AI due to AIA [15].

#### Type of AI

There were only two studies that reported an association between the type of AI and the incidence of AIA, and they had mixed results. One study reported a higher incidence of AIA with steroidal AIs when compared with non-steroidal AIs [36], while another study reported a higher risk of AIA with letrozole [16].

#### Genetic factors

One study found that single nucleotide polymorphisms were located in genes involved in the metabolism of oestrogens and vitamin D. In particular, the *CYP17A1* and *VDR* genes were significantly associated with a higher incidence of AIA, and the *CYP27B1* gene was related to AI discontinuation [31]. Another study reported a greater incidence of AIA and pain severity with patients carrying the G allele of rs2073618 in the osteoprotegerin gene [37].

### Protective factors against AIA

#### Vitamin D level

There were mixed findings regarding the effect of vitamin D level on AIA (Table 4). Three studies reported that having vitamin D3 levels ≥40 ng/ml was associated with attenuated joint pain or lower risk of AIA [38, 41, 43], while three studies did not find any significant association of AIA with vitamin D3 levels [39, 40, 42]. One study also reported that patients with the *VDR* Fok I variant genotype (vitamin D receptor polymorphism) were less likely to develop AIA than those with the wild-type VDR [42].

**Table 4.** Protective factors for AIA

Risk factor	Preventive factor	Subjects on AI, *n*	Type of study	Reference
Vitamin D level	Lower joint pain intensity in patients with 25(OH)D levels ≥40 ng/ml when compared with 25(OH)D levels <40 ng/ml (*P* = 0.02)	290	PO	Prieto-Alhambra and Javaid, 2011 [38]
	No difference in the incidence of AIA between high-dose 50 000 IU vitamin D3 supplement group and 800 IU vitamin D3 supplement group. Neither baseline vitamin D nor 12-week vitamin D level was predictive of AIA (*P* = 0.449)	*N* = 93; 800 IU daily vitamin D3 = 47, 50 000 IU weekly, then 2000 IU daily vitamin D3 = 46	RCT	Niravath *et al*., 2019 [39]
	No difference in the incidence of AIA between vitamin D3 supplement and placebo groups according to CPIS (*P* = 0.069). However, lower incidence of AIA in the vitamin D3 supplement group was found according to post hoc analysis using BPI (*P* = 0.024)	*N* = 160; 30 000 IU/week vitamin D3 = 80, placebo = 80	RCT	Khan *et al*., 2017 [40]
	Lower incidence and attenuated AIA in patients with 25(OH)D level ≥40 ng/ml (*P* = 0.02)	260	PO	Prieto-Alhambra and Javaid, 2011 [41]
	Vitamin D levels (*P* = 0.88) was not associated with the development of AIA. Lower likelihood of AIA in patients with VDR Fok I variant genotype compared with those with wild-type VDR (*P* < 0.0001)	*N* = 216; arthralgia = 72, controls = 144	RO	Niravath *et al*., 2018 [42]
	Lower likelihood of AIA in patients with baseline vitamin D levels >40 ng/ml (*P* = 0.037)	51	PO	Singer *et al*., 2014 [43]
Previous treatment	Lower incidence of AIA in patients who had prior endocrine therapy (SERM, AI or LH-RH agonist; *P* < 0.05)	329	RO	Horimoto *et al*., 2009 [18]
	Lower incidence of AIA in patients on chronic diuretic therapy (*P* = 0.01)	*N* = 288; diuretics = 42, controls = 246	RO	Xepapadakis *et al*., 2010 [44]
	Lower rate of AIA in patients who received tamoxifen prior to AIs or received AIs plus calcium or bisphosphonates (*P* < 0.001)	316	RO	Muslimani *et al*., 2009 [36]

25(OH)D: 25-hydroxyvitamin D; CPIS: Categorical Pain Intensity Scale; LH-RH: luteinizing hormone–releasing hormone.

#### Previous treatments

Prior endocrine therapy (e.g. selective oestrogen receptor modulators, luteinizing hormone–releasing hormone receptor agonists or another AI), chronic diuretic therapy and concurrent treatment with calcium or bisphosphonates were associated with lower rates of arthralgia [18, 36, 44].

### AIA management

#### Over-the-counter medications

Table 5 reflects studies that showed no significant benefit with omega-3 supplements when considering all patients with AIA, but a possible benefit among obese patients [45, 46]. Vitamin B12 supplements were associated with a reduction in pain scores among patients with AIA [48]. However, vitamin D3 supplements were not associated with improvement of symptoms [49]. One patient in a case report had relief of symptoms with melatonin exposure via light-emitting diodes mounted on a cap [47].

**Table 5.** Treatments for AIA

Treatment		Intervention	Outcome	Subjects with AIA, *n*	Type of study	Reference
OTC medications		O3-FAs 3.3 g daily for 24 weeks	No significant difference in BPI-SF scores between O3-FA and placebo groups (*P* = 0.58)	*N* = 249; O3-FA = 122, placebo = 127	RCT	Hershman *et al*., 2015 [45]
		O3-FAs 3.3 g daily for 24 weeks	Lower BPI worst pain scores (*P* = 0.02), average pain and pain interference scores (*P* = 0.01) at 24 weeks with O3-FA use in obese patient group	*N* = 249; non-obese = 139, obese = 110	RO (post hoc analysis of SWOG S0927 RCT)	Shen *et al*., 2018 [46]
		Melatonin/light-emitting diodes mounted on a cap	Decreased morning stiffness and suppression of joint pain with extended use	1	CR	Burk, 2008 [47]
		2500 μg of sublingual vitamin B12 daily for 90 days	Improved average pain (*P* < 0.0001) and worst pain scores (*P* = 0.0003) at 3 months	41	PO (single arm)	Campbell *et al*., 2018 [48]
		600 IU D3 or 4000 IU D3 daily for 6 months	No significant differences in BCPT-MS scores between 600 IU D3 and 4000 IU D3 groups at 6 months	*N* = 116; 4000 IU D3 = 57, 600 IU D3 = 59	RCT	Shapiro *et al*., 2016 [49]
Prescribed medications		Duloxetine 30 mg once daily for 1 week, twice daily for 11 weeks, then once daily for 1 week	Lower average joint pain score in the duloxetine group (*P* = 0.0002)	*N* = 299; duloxetine = 150, placebo = 149	RCT	Henry *et al*., 2018 [50]
		Oral prednisolone 5 mg once daily for 1 week	Improved joint pain in 67% of patients immediately, 63% at 1 month and 52% at 2 months	29	PO (single arm)	Kubo *et al*., 2012 [51]
		Two surgically implanted pellets containing testosterone 120 mg and anastrozole 8 mg; later topical application of testosterone 10.4 mg	No significant difference in BPI average joint pain scores between testosterone and placebo groups at 3 (*P* = 0.50) or 6 months (*P* = 0.67)	*N* = 227; testosterone = 114, placebo = 113	RCT	Cathcart-Rake *et al*., 2021 [52]
		Thymosin α1 1.6 mg, twice a week for 4 weeks	Improved mean BPI-SF worst pain score (*P* < 0.001), pain severity (*P* = 0.01), pain-related functional interference (*P* < 0.001) and WOMAC score (*P* < 0.001)	16	PO (single arm)	Zhang *et al*., 2010 [53]
		Glucosamine sulphate (1500 mg/day) and chondroitin sulphate (1200 mg/day) for 24 weeks	At week 24, improved joint function and pain according to WOMAC and M-SACRAH and improved pain interference according to BPI (all *P* < 0.05)	53	PO (single arm)	Greenlee *et al*., 2013 [54]
CAM	Acupuncture	Sham or real EA twice weekly for 6 weeks	No significant difference in joint stiffness and function according to WOMAC and overall pain severity and interference according to BPI-SF between sham and real EA groups	*N* = 29; real EA = 14, sham EA = 15	PO	Oh *et al*., 2013 [55]
		12 true acupuncture or sham acupuncture sessions over 6 weeks	Improved BPI-SF worst pain scores (*P* < 0.001), pain severity (*P* = 0.003) and pain-related interference (*P* = 0.002) in TA group when compared with SA group at 6 weeks	*N* = 43; TA = 23, SA = 20	RCT	Crew *et al*., 2010 [56]
		12 acupuncture sessions over 6 weeks followed by 1 session per week for 6 weeks	Greater reduction in BPI-WP pain scores in TA group when compared with SA or control group at 6 weeks (*P* = 0.01)	*N* = 226; TA = 110, SA = 59, waitlist controls = 57	RCT	Hershman *et al*., 2018 [57]
		12 acupuncture sessions over 6 weeks followed by 1 session per week for 6 weeks	Lower BPI-WP scores in TA when compared with SA or controls at 52 weeks (*P* = 0.01 and *P* = 0.03, respectively)	*N* = 226; TA = 110, SA = 59, waitlist controls = 57	RCT	Hershman *et al*., 2022 [58]
		10 acupuncture sessions over 8 weeks	Greater reduction in BPI mean pain severity in EA group when compared with the control group at week 8 (*P* = 0.0004) and week 12 (*P* < 0.0001). Similar outcomes in SA group at weeks 8 and 12 (*P* < 0.001 and *P* = 0.0036, respectively). No statistical difference in outcomes between EA and SA groups at both time points	*N* = 67; EA = 22, SA = 22, waitlist controls = 23	RCT	Mao *et al*., 2014 [59]
		Auricular point acupressure treatment each week for 4 weeks by a trained therapist	Decrease in worst pain (50%) and pain interference (42%) according to BPI-SF and improvement in joint function (31%) and stiffness (28%) according to WOMAC	20	PO (single arm)	Yeh *et al*., 2017 [60]
		8–12 acupuncture sessions over 4 weeks	Improved pain severity score, relief of treatment-related pain, and pain interference according to BPI and improved joint pain, stiffness and physical function according to WOMAC	8	PO (single arm)	Kim and Kang, 2019 [61]
	Others	Emu oil rubbed into skin of affected joints three times daily for 8 weeks	Improvement in VAS, BPI pain and interference scores (*P* < 0.001) at 8 weeks when compared with baseline for both emu and placebo oil groups but no significant difference in pain scores between the groups	*N* = 87; emu oil = 43, placebo oil = 44	RCT	Chan *et al*., 2017 [62]
		Kampo therapy (Japanese traditional medicine); consumption of powdered processed aconitine root 3.0 g/ day	Complete resolution of AIA	1	CR	Chino *et al*., 2011 [63]
		*Ruta graveolens* 5CH and *Rhus toxicodendron* 9CH (5 granules, twice a day) up to 7 days before starting AI and continued for 3 months	Greater reduction in joint pain scores in homeopathy group when compared with controls at 3 months (*P* = 0.0001)	*N* = 40; homeopathy = 20, controls = 20	PO	Karp *et al*., 2016 [64]
		CAM (vitamins, high-dose vitamin C, food supplements, mistletoe, enzymes, acupuncture, homeopathy, Chinese herbs/tea, mushrooms, meditation, prayer, relaxation techniques, yoga, tai chi, qigong, or bioresonance)	No significant difference in pain between CAM and control groups	*N* = 1396; CAM = 901, controls = 495	PO	Hack *et al*., 2020 [65]
		Consumption of tart cherry concentrate in water daily for 6 weeks	Greater decrease in pain in tart cherry group (*P* = 0.034)	*N* = 48; tart cherry = 23, placebo = 25	RCT	Shenouda *et al*., 2021 [66]
Physical activity		Yoga first group: 6 weeks of yoga, 2 weeks of rest, then 6 weeks of massage Massage first group: 6 weeks of massage, 2 weeks of rest, then 6 weeks of yoga	Greater decrease in WOMAC pain score in yoga first group when compared to massage first group (*P* < 0.001)	*N* = 60; yoga first = 30, massage first = 30	RCT	Tsai *et al*., 2021 [67]
		Yoga sessions twice a week for 8 weeks	Reduction in pain severity (*P* < 0.05)	10	PO (single arm)	Galantino *et al*., 2012 [68]
		Lyengar yoga classes twice weekly for 12 weeks	Reductions in hand stiffness (AUSCAN), knee/hip pain, stiffness and impaired function (WOMAC), overall BPI pain severity and interference (BPI) (*P* < 0.05)	10	PO (single arm)	Jacobsen *et al*., 2015 [69]
		8-week home-based program combining upper and lower body resistance exercises with self-selected aerobic exercises	Improvement in arthritic pain post- intervention (*P* = 0.01)	26	PO (single arm)	DeNysschen *et al*., 2014 [70]
		Walk With Ease (WWE): goal of minimum 30 min of walking 5 days/week (150 min/week) for 6 weeks	Increased walking minutes per week (*P* < 0.01), improved WOMAC stiffness score (*P* < 0.05), greater decrease in worst joint pain scores, pain severity and pain interference scores (*P* < 0.001) in the intervention group when compared to control group at 12 months	*N* = 62; walking = 31, waitlist controls = 31	RCT	Nyrop *et al*., 2017 [71]
		A year-long program consisting of a combination of twice a week supervised resistance training and a home-based aerobic exercise program of 150 min/week	Greater decrease in worst joint pain scores (*P* < 0.001) and pain severity and interference (*P* < 0.001) in the exercise group compared with the usual care group at 12 months	*N* = 121; exercise = 61, usual care = 60	RCT	Irwin *et al*., 2015 [72]
		TaiChi4Joint, remotely delivered tai chi classes for 12 weeks	Improvement in BPI (*P* < 0.001), AUSCAN pain subscale (*P* = 0.007) and AUSCAN function subscale (*P* = 0.004) scores at 3 months compared with baseline	22	PO (single arm)	Gomaa *et al*., 2022 [73]
Switch to different AI		Switch to letrozole	Resolution of symptoms and long-term adherence	1	CR	Bryce *et al*., 2012 [74]
		Three patients switched to a different AI	Improvement in symptoms in 67% of subjects after the switch	42	PO	Boonstra *et al*., 2013 [17]
		Two patients switched to a different AI and six patients switched to a SERM	Improved symptoms in 50% of patients who switched to AI and 83% of patients who switched to SERM	90	RO	Horimoto *et al*., 2009 [18]
		Nineteen patients switched to a different AI	Reduction in the severity of AIA in all patients but not complete resolution	62	RO	Moscetti *et al*., 2015 [34]

CR: case report; OTC: over the counter; BPI-SF: Brief Pain Inventory–Short Form; O3-FA: Omega-3 fatty acid; BCPT-MS: Breast Cancer Prevention Trial–Musculoskeletal Symptom Scale; M-SACRAH: Modified Score for the Assessment of Chronic Rheumatoid Affections of the Hands; EA: electroacupuncture.

#### Prescribed medications

Treatment with oral prednisolone, duloxetine, glucosamine sulphate, chondroitin sulphate and thymosin were all significantly associated with improvement of AIA [44, 50, 51, 53, 54] (Table 5). The largest study assessing AIA medical treatment was a prospective randomized trial comparing the use of duloxetine 30 mg on a 13-week treatment regimen to placebo and yielded significantly lower average joint pain scores in the duloxetine cohort compared with the placebo cohort (*P* = 0.0002) [50]. The next largest study was a prospective observational study assessing the use of glucosamine sulphate (1500 mg/day) and chondroitin sulphate (1200 mg/day) for 24 weeks, resulting in significant improvement in joint pain and function at the end of the intervention (*P* < 0.05) [54]. A randomized trial was conducted investigating the effects of testosterone use, but neither subcutaneous nor topical administration was associated with a significant change in symptoms [52].

#### Complementary and alternative medicine (CAM)

While many forms of alternative medicine have been investigated as treatment for AIA, the most robust data have been found for acupuncture. Four RCTs showed a significant reduction in AIA symptoms with acupuncture sessions [56–59]. One study was done on auricular point acupressure, a type of acupuncture that does not use needles, which also improved symptoms [60]. Other therapies described were Kampo therapy (Japanese traditional medicine) [63], *Ruta graveolens* and *Rhus toxicodendron* (types of homeopathic medicines) [64] and tart cherry concentrate [66], which all resulted in either complete resolution or a reduction of AIA symptoms. One study reported significant improvement in pain with massaging topical oil onto affected joints [62]. However, a study that looked at the effect of all CAM treatments, not a particular treatment, did not find any difference in pain between the control and CAM groups [65].

#### Physical activity

Yoga, exercise (walking, aerobic exercises, resistance exercises) and tai chi were all associated with a reduction in AIA symptoms [67, 68, 70–73, 75].

#### Switching to a different AI

None of the observational studies or RCTs included in this review directly studied the effect of switching to a different AI. However, in the prospective study done by Boonstra *et al*. [17], two of three patients who switched to a different AI due to AIA experienced an improvement of symptoms, while in the retrospective study done by Moscetti *et al*. [34], all 19 patients who switched to a different AI experienced alleviation but not complete resolution of symptoms. In one case report, a patient experienced resolution of AIA symptoms after switching from anastrozole to letrozole and reported long-term adherence afterwards [74].

#### Relationship between AIA and cancer outcome

In a retrospective study of 1502 patients, patients who experienced AIA symptoms had significantly improved overall survival (*P* < 0.01) and cancer-free survival (*P* < 0.001) than patients who did not report AIA symptoms [22].

## Discussion

AIAs are common, occurring on average in up to 40% of patients, leading to increased symptom burden, drug non-adherence and discontinuation of therapy. Although there are a limited number of studies, most cases were non-inflammatory in character. A shorter time since the last menstrual period [25, 32] and pre-existing joint pain [13, 16, 21, 26] have been consistently associated with a higher risk of developing AIA. Prior exposure to certain medications or therapies, including pain medications, endocrine therapies, chemotherapy and radiation, has also been reported to be associated with AIA development [16, 33]. A few studies described preventative factors, including serum vitamin D3 levels, but results are inconsistent and larger studies are needed. Among treatment options, duloxetine, acupuncture and exercise have been associated with decreased arthralgia and are supported by RCTs. There is no consensus regarding the relationship between AIA development and tumour outcomes [22, 76, 77].

The exact mechanism of AIA remains unclear, but it is likely that more than one pathway is involved. Most mechanisms involve an oestrogen deprivation state [78]. Oestrogen is known to suppress inflammatory cytokine production. Similar to arthralgia that manifests in perimenopausal women, AIA may be caused by oestrogen deprivation, leading to the production of inflammatory cytokines in joint chondrocytes [79]. Moreover, a reduction of antinociceptive effects of oestrogen in a low-oestrogen state may lower the pain threshold in patients, making them more susceptible to developing arthralgia [80]. Accelerated bone loss from oestrogen deprivation likely also contributes to AIA and explains the higher incidences of osteopenia and osteoporosis found in AIA [81]. Lastly, given the higher incidences of AIA in those with a shorter time since the last menstrual period [25, 32], rapid fluctuation in the oestrogen level rather than the absolute oestrogen level may contribute to AIA.

Based on the review and potential mechanisms, below are our proposed recommendations. Educating patients prior to the start of AIs can encourage greater adherence and empower patients to be more vigilant about their potential toxicity. Although high-powered prospective studies are still lacking, there are data that identify some modifiable risk factors (high BMI, pre-existing joint pain) that may be addressable prior to the start of AI treatment to reduce the chances of AIA [17, 29]. Patients may be additionally motivated to seek out healthier eating and/or exercise if they know that a higher BMI may contribute to AIA development.

The chances of inflammatory arthropathy with AI treatment are very low; physical exam findings and laboratory or imaging results should be carefully assessed for the presence of inflammation, as a lack of inflammation may help avoid deleterious side effects of inappropriately prescribed modalities such as systemic steroids. In those with AIA, exercise or physical therapy should be offered as the first line of therapy. Various over-the-counter agents such as acetaminophen and NSAIDs can be used as adjuncts for pain management. If pain is still poorly controlled, we recommend trialling duloxetine as long as there are no contraindications. Vitamin D supplements should be recommended (being low risk when taken in recommended doses). A DXA scan should be done, if not done already, and bisphosphonates for patients with osteopenia or osteoporosis should be offered if appropriate [38, 41, 43]. Switching to an alternate AI is a good therapeutic strategy, as numerous studies have reported on the resolution or attenuation of AIA symptoms after switching to a different AI [82]. Additionally, research has shown better cancer outcomes with AIs *vs* tamoxifen, so we recommend alternate AIs before switching to tamoxifen [2, 7]. Lastly, regular follow-ups are crucial for monitoring pain control and ensuring compliance to AI treatment.

## Future directions

Further study is needed for both assessment and management of AIAs. Translational studies to elucidate serologic markers as well as synovial tissue analysis of the affected joint(s) can yield a better understanding of AIA pathophysiology and further assist in identifying potential subcategories of AIA. Additionally, identifying validated tools to properly characterize AIAs and creating a unified terminology with consistent methods of assessment will facilitate future clinical trials. There is a paucity of reliable AIA management options and preventative therapies. Various supplements and prescribed medications have been suggested, but RCTs should be performed to validate their efficacy. All in all, AIA affects a significant portion of patients on AI therapy, with the severity limiting treatment adherence in some patients. An improved understanding of the diagnosis and management of this disorder is necessary to improve patient tolerance and quality of life.

## Data Availability

Raw data were generated at the University of Chicago Medical Center. Derived data supporting the findings of this study are available from the corresponding author upon request.
